# Understanding Atrial Fibrillation in the Context of Body Fat Distribution∗

**DOI:** 10.1016/j.jacadv.2024.100972

**Published:** 2024-05-07

**Authors:** Kamala P. Tamirisa, Prashanthan Sanders, Amin Al-Ahmad

**Affiliations:** a Texas Cardiac Arrhythmia, Dallas/Austin, Texas, USA; b Centre for Heart Rhythm Disorders, University of Adelaide, and Royal Adelaide Hospital, Adelaide, Australia

**Keywords:** atrial fibrillation, body fat

**Figure:**
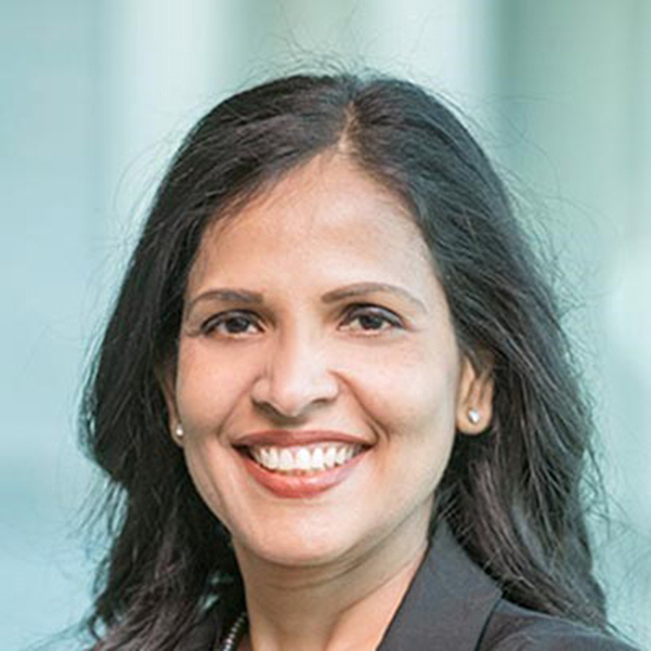

Atrial fibrillation (AF) is the most prevalent arrhythmia globally, affecting 60 million people, and its burden is predicted to increase by 60% in 2050.^1^ Obesity is a significant predictor of AF, with studies showing a 30% increase in AF for every 5-unit increase in body mass index.^2^^,^^3^ The ongoing Framingham Heart Study has shown that obesity increases AF risk by 50%.^4^ Obesity rates have doubled since 1990, and over 1 billion people are projected to be obese by 2025.^5^

Obesity frequently coexists with known risk factors for AF.^6^ It increases the risk of various comorbidities, including diabetes, hypertension, and heart failure, all of which contribute to AF pathogenesis, leading to worse outcomes.^1^^,^^7^ Additionally, obesity directly contributes to atrial structural and electrical remodeling, which are implicated in AF pathology.^8^ Epicardial adipose tissue distribution and the release of pro-inflammatory cytokines leading to atrial remodeling, altered action potential duration, and fibrosis play a crucial role in AF pathogenesis.^7^

Rhythm control using catheter ablation (CA) has been shown to improve outcomes in patients with AF compared to anti-arrhythmic medications or rate control strategies.^9^ AF-free survival post-CA varies widely among individuals and left atrial (LA) remodeling is often used as a surrogate marker for recurrent AF post-CA.^10^ Obese patients have increased rates of AF recurrence post-CA, which can be improved with weight loss.^11^ However, it is interesting to note that even the nonobese patients have recurrent AF post-CA after a 1-year period, suggesting that there may be multiple factors playing a key role.^12^ The association of body fat distribution in nonobese patients and LA reverse remodeling (LARR) post-CA is largely unclear. To answer this question, the authors highlighted the interplay of body fat distribution type, LARR, and AF recurrence post-CA.

The study by Hirose et al included 116 consecutive nonobese paroxysmal and persistent AF patients (75% men) who underwent CA between 2020 and 2022 at the University of Tokyo Hospital. Patients with obesity (body mass index of ≥30 kg/m^2^) were excluded.^13^ Various body size metrics and inflammatory markers (interleukin-6 and C-reactive protein) were measured. Waist/hip ratio (W/H ratio), bio-impedance analysis using a body composition analyzer (Tanita Body Composition Analyzer), body fat percentage, and central fat percentage (CF%) were used to assess the amount and type of body fat distribution. Transthoracic echocardiograms were performed pre- and 6 months post-CA, and LA mass/volumes were indexed for body surface area. Recurrence of >30-second AF after a 2-month blanking period was evaluated using a 12-lead electrocardiogram and 24-hour Holter monitoring. LARR was defined as a >15% reduction in the LA volume index at 6 months. 62% of patients underwent radiofrequency ablation, 37% underwent cryoablation, and 26% had additional CA beyond pulmonary vein isolation.

Fifty-one percent of patients had LARR based on echocardiographic measurements post-CA. Six months post-CA, the baseline group with a higher W/H ratio and CF% showed persistent LA enlargement compared to their peers (W/H ratio: 35.2 ± 11.6 mL/m^2^ vs 29.4 ± 8.4 mL/m^2^, *P* = 0.005; CF%: 35.2 ± 10.6 mL/m^2^ vs 29.5 ± 9.8 mL/m^2^, *P* = 0.003). In multivariate analysis, a higher W/H ratio and CF% were associated with a lack of LARR (adjusted odds ratios of 3.86 and 2.82 per 0.10 and 10% increase with a *P* value <0.01). The combined assessment of CF% with W/H ratio risk stratified for persistent LA enlargement. Patients with higher W/H ratios, body fat percentage, and CF% had increased levels of inflammatory markers. The main conclusion is that central adiposity (higher W/H ratio) and higher CF% are associated with a lack of LARR post-CA in nonobese patients.

The optimal method to quantify obesity remains elusive. In this regard, the current study provides greater granularity to the link between fat distribution and AF. While there is data on the multifactorial effects of obesity on AF pathogenesis and post-CA outcomes, it is largely unknown whether a specific pattern of body fat distribution can predict LARR and recurrent AF post-CA. This study examines LARR and recurrent AF post-CA in nonobese patients and the association of body fat distribution. There was no correlation between LA dimensions and independent body size metric measures at baseline preprocedure. Post-CA, a higher W/H ratio and CF%, were associated with persistent LA enlargement, but the relationship was attenuated by multivariable adjustments, suggesting complex interrelated factors may play a role. CA patients with an independent higher W/H ratio and CF%, as well as combined W/H ratio with CF% were associated with reduced LARR, independent of baseline LA size and AF type. These findings are consistent with the data that links the presence of epicardial fat, an established feature of central obesity, with AF and AF recurrence post-CA.^14^ Epicardial fat has emerged as a burgeoning area of interest, linking weight with the mechanisms of atrial remodeling and AF.^15^ Critical to this link is the role of centrally located fat in secreting pro-inflammatory markers associated with atrial fibrosis formation, a substrate for AF.

Limitations of the study include the predominance of the male study population and the geographical isolation (Japan only), potentially limiting generalizability. There is limited information on the spectrum of lifestyle and risk factors that are known to be associated with AF recurrence.^16^ Incomplete information on the subdistribution of fat could have impacted the analysis. The preprocedural AF burden was not clearly stated, and postprocedure follow-ups relied on a 24-hour Holter monitor, electrocardiogram, and single post-CA echocardiogram, possibly leading to underdiagnosis of post-CA AF recurrence and affecting LA remodeling assessment.

Understanding the relationship between body fat distribution and AF recurrence post-CA is crucial for improving overall outcomes in both obese and nonobese populations. Studying different body types, such as pear-shaped vs apple-shaped, could provide valuable insights into the relationship between body fat distribution and AF recurrence. This research could inform the development of targeted interventions aimed at reducing central obesity, such as tailored lifestyle interventions, dietary modifications, and/or exercise regimens to improve AF outcomes post-CA. Given the current interest in weight loss therapy, bariatric surgery, and novel glucagon-like peptide-1 antagonists, it would be intriguing to investigate if there is any correlation between fat distribution and the recurrence of post-CA AF.

## Funding support and author disclosures

Dr Tamirisa is a speaker for Abbott and Sanofi; and is on the advisory boards of Boston Scientific, CathRx, Medtronic, Abbott Medical, and Pacemate. Dr Sanders reports that the University of Adelaide has received, on his behalf, lecture and/or consulting fees from Medtronic, Boston Scientific, Abbott Medical, Pacemate, and CathRx; and reports that the University of Adelaide has received, on his behalf, research funding from Medtronic, Abbott Medical, Boston Scientific, Pacemate, and Becton Dickinson. Dr Al-Ahmad has received consulting and speaking for Medtronic, Boston Scientific, Abbott, and Biosense Webster.
